# A Phase I Trial of Pox PSA vaccines (PROSTVAC^®^-VF) with B7-1, ICAM-1, and LFA-3 co-stimulatory molecules (TRICOM™) in Patients with Prostate Cancer

**DOI:** 10.1186/1479-5876-4-1

**Published:** 2006-01-03

**Authors:** RS DiPaola, M Plante, H Kaufman, DP Petrylak, R Israeli, E Lattime, K Manson, T Schuetz

**Affiliations:** 1 The Cancer Institute of New Jersey, University of Medicine and Dentistry of New Jersey, New Brunswick, NJ, USA; 2 University of Vermont, South Burlington, VT, USA; 3 Columbia Presbyterian Medical Center, New York, NY, USA; 4 Staten Island Urological Research, Staten Island, NY, USA; 5 Therion Biologies Corporation, Cambridge, MA, USA

**Keywords:** PSA, Prostate specific antigen, vaccine, co-stimulatory molecules

## Abstract

**Purpose:**

Based on previous studies that demonstrated the safety profile and preliminary clinical activity of prostate specific antigen (PSA) targeted therapeutic vaccines, as well as recent laboratory data supporting the value of the addition of co-stimulatory molecules B7-1, ICAM-1, and LFA-3 (designated TRICOM™) to these vaccines, we conducted a Phase I study to evaluate the safety and immunogenicity of a novel vaccinia and fowlpox vaccine incorporating the PSA gene sequence and TRICOM.

**Methods:**

In this study, ten patients with androgen independent prostate cancer with or without metastatic disease were enrolled. Patients were treated with 2 × l0^8 ^pfu of a recombinant vaccinia virus vaccine (PROSTVAC-V) followed by 1 × 10^9 ^pfu of the booster recombinant fowlpox virus (PROSTVAC-F) both with gene sequences for PSA and TRICOM. The mean age of patients enrolled in the study was 70 (range 63 to 79). The mean PSA at baseline was 434 (range 9 – 1424).

**Results:**

There were no deaths, and no Grade 3 or 4 adverse events. The most commonly reported adverse events, regardless of causality, were injection site reactions and fatigue. One serious adverse event (SAE) occurred that was unrelated to vaccine; this patient developed progressive disease with a new sphenoid metastasis. PSA was measured at week 4 and week 8. Four patients had stable disease (with less than 25% increase in PSA) through the week 8 study period. Anti-PSA antibodies were not induced with therapy: however, anti-vaccinia titers increased in all patients.

**Conclusion:**

This study demonstrated that vaccination with PROSTVAC-V and PROSTVAC-F combined with TRICOM is well-tolerated and generated an immune response to vaccinia. Therefore, PROSTVAC-VF/TRICOM represents a feasible therapeutic approach for further phase II and III study in patients with prostate cancer.

## Introduction

In 2005, approximately 230,000 new patients were diagnosed with prostate cancer, and about 30,000 died, mostly from metastatic disease [1]. Most patients in the US are diagnosed with localized or regional disease that is treated with surgery (e.g., prostatectomy) and/or radiation therapy, including brachytherapy. Despite local treatment, approximately 50% of patients develop recurrent disease. These patients are generally treated with androgen blockade, which improves pain, and urinary symptoms, but is only temporarily effective. Two recently published phase III studies utilizing taxane-based therapy for androgen-independent prostate cancer have demonstrated an improvement in survival [2,3]. Despite this improvement in therapy, the majority of men will die from metastatic disease, thus highlighting the need for novel therapeutic approaches.

Harnessing the immune system to identify and destroy cancer cells, or immunotherapy, is one such novel approach. Initial efforts to develop an immune-mediated treatment for patients with prostate cancer included the study of pox virus vaccination. In prostate cancer, Eder et al. treated patients with recombinant vaccinia PSA (rV-PSA) vaccine and demonstrated safety [4]. Gulley et al. recently completed a Phase I clinical trial of rV-PSA in 42 patients with metastatic androgen independent prostate cancer also demonstrating safety and immunological response [5]. Our prior study demonstrated the safety and activity of the combined approach of fowlpox and vaccinia PSA vaccines without co-stimulatory molecules in a randomized Phase II trial; this study demonstrated the safety of this approach and suggested the sequence of vaccinia followed by fowlpox was clinically superior [6].

A number of additional co-stimulatory molecules on antigen presenting cells have been identified including ICAM-1, B7.1, and leukocyte function associated antigen-3 (LFA-3). Constructs using poxviral vectors (fowlpox and vaccinia) have been generated that contain this triad of co-stimulatory molecule transgenes (designated TRICOM). Preclinical studies using TRICOM constructs have shown that they are superior to those constructs that contain only one or two of the co-stimulatory molecules [7,8]. Phase I studies have been completed combining CEA-based pox virus vaccines with TRICOM demonstrating the feasibility of this approach [9].

This preliminary data provided the justification for a phase I study to evaluate the safety of vaccinia and fowlpox PSA vaccines in combination with TRICOM. Although poxvirus vaccines expressing PSA and TRICOM had been previously evaluated in separate trials, the clinical trial reported here used both in a single vector and was designed as a safety/feasibility trial. The dose of the vaccine was selected based on prior studies demonstrating an acceptable safety profile [6,9].

## Materials and methods

### Patient eligibility

Men age ≥ 18 yrs with prior small pox immunization were eligible. The patients had an ECOG performance status of ≤ 2 with life expectancy of at least 6 months. The patients must have had histologically-proven adenocarcinoma of the prostate with hormone refractory disease with rising PSA or with metastatic disease. Patients progressing on androgen ablation therapy must have been maintained on LHRH agonist and be off flutamide or bicalutamide for 4 or 6 weeks respectively. Patients who received any prior chemotherapy were not eligible for this trial. Patients had to have adequate liver, hematopoetic and renal function (Hgb>8 g/dl, WBC>2000 cells/mm3, platelet>100,000 cells/mm3, bilirubin<1.5 times ULN, serum creatinine< 2 mg/dl).

### Treatment

All patients signed institutional review board (IRB)-approved informed consent forms prior to undergoing screening procedures. On day 1, after study personnel completed the review of eligibility criteria, baseline physical examination, measurement of vital signs, review of local laboratory assessments (including PSA), and blood sampling for immunologic analysis, patients received 1 dose of PROSTVAC-V (the vaccinia virus with gene sequences for PSA and TRICOM as described below). On day 29, after study personnel completed physical examination, vital sign measurements, review of laboratory values (including PSA), blood sampling for immunologic analysis, and assessment of adverse events (AEs) and concomitant therapy, patients received 1 dose of PROSTVAC-F (the fowlpox virus with gene sequences for PSA and TRICOM as described below). On day 43, study personnel were to interview patients by telephone to document AEs. On day 57, patients were to return to the clinic for the study termination visit, including a physical examination, vital sign measurements, Eastern Cooperative Oncology Group Performance Status (ECOG), electrocardiogram (ECG), laboratory assessments (including PSA), blood sampling for antibody and immunologic analysis, and assessment of AEs and concomitant therapy.

### Vaccine preparation

PROSTVAC-VF consists of two genetically engineered vaccines (recombinant vaccinia or fowlpox virus) administered in a sequential regimen. The vaccinia strain used in PROSTVAC-V is a partially attenuated version of the virus used for smallpox immunization. Fowlpox is unable to replicate in human cells but has been shown to be an effective means of boosting cellular immune responses initiated with vaccinia. The viral vectors are engineered to contain the gene encoding PSA which contains an alteration in the HLA-A2 specific epitope that is designed to enhance the immunogenicity of the expressed antigen. In addition, these viruses both contain the genes encoding three co-stimulatory molecules, B7.1, ICAM-1 and LFA-3. The vaccines are a clarified lysate of pox virus-infected primary chicken embryo dermal cells that has been partially purified. The product is re-suspended in phosphate-buffered saline with 10% glycerol and stored at -70 degrees C or colder. Virus is vialed at 0.3 ml/vial at a target concentration of ≥ 10^9 ^plaque forming units (pfu)/ml. On the day of administration the vial is thawed at room temperature and diluted with 0.9% sodium chloride for injection using a 22 gauge needle. The dose of vaccine was based on safety data from previous poxvirus vaccine trials evaluating poxviruses expressing PSA and poxviruses expressing TRICOM with CEA.

### Patient assessment

The current study was a phase I trial with assessment of dose limiting toxicity (DLT) at this single planned dose level, as defined by any grade 2 or greater allergic reaction of asymptomatic bronchospasm and/or generalized urticaria, or autoimmune response; or any grade 3 or greater hematologic or non-hematologic adverse event that is attributable to vaccine. Although assessment of safety was the primary end point, we also reported biochemical progression as an increase in PSA of greater than 25% from baseline, and stable disease as a PSA increase of ≤ 25% and decrease <50%. Patients were considered to have a response if they experienced ≥ 50% decrease in PSA.

### Antibody measurement

Anti-vaccinia, anti-fowlpox, and anti-PSA antibody response were evaluated using ELISA specific for vaccinia, fowlpox, and PSA. Ninety six well microtiter plates were coated overnight with PSA protein from human seminal fluid (Biodesign), parental vaccinia lysate (TBC-Wyeth), or parental fowlpox lysate (TBC-FPV). The next day the plates were blocked, washed and two-fold serial dilutions of patient sera, controls, and naïve sera were added to the appropriate wells in duplicate. Following an overnight incubation binding antibody was detected using a conjugated anti-IgG secondary antibody and the color developed with the appropriate substrate. The ELISA plates were analyzed using a Molecular Devices SpectraMax^® ^plate reader.

The titer was determined for each sample. The titer is defined as the reciprocal of the dilution with an absorbance of three times the absorbance of the appropriate control, which is tested at a single dilution. Human naïve vaccinia and human naïve fowlpox sera were used as controls for the poxvirus ELISAs. Each patient's pre-vaccination serum sample was used as the control for the PSA ELISA. A positive response was defined as a two-fold increase of the post-immunization sample compared to the pre-immunization sample.

### Statistical methods

The sample size was chosen to provide an adequate initial demonstration of safety and tolerability. Immune and PSA response data was summarized as a mean value for all patients at each time point. The planned dose was to be considered the recommended phase II dose if < 2 patients out of 10 had a DLT. Further dose escalation was not felt to be necessary, given prior studies demonstrating immune effects using similar dosing without TRICOM.

## Results

### Patient demographics

A total of 10 men with androgen independent prostate cancer with biochemical only or objective progression were enrolled (Table 1). Patients received one dose of PROSTVAC-V (vaccinia virus) followed by a booster dose of PROSTVAC-F (fowlpox virus) after 4 weeks. The mean age of patients enrolled in the study was 70 (range 63 to 79). Nine patients were Caucasian and one was African American. The mean PSA at baseline was 434 (range 9 – 1424). The mean anti-vaccinia titer at baseline was 700 (range 200–1600). The patients were followed up to 8 weeks after the initial dose.

**Table 1:** Patient Demographics

**Age (years)**	
n	10
Mean	69.6
Range	63–79

**Race/Ethnicity**	

n	10
Caucasians	9
African Americans	1

**Baseline PSA (ng/mL)**	

n	10
Mean	434
Range	9–1424

**Baseline Anti-Vaccinia Titer**	

n	10
Mean	700
Range	200–1600

### Toxicity

No patients experienced dose limiting toxicity and there were no deaths on study. Nine patients reported 37 adverse events. There were no Grade 3 or 4 adverse events. The most commonly reported adverse events, regardless of causality, were injection site reactions and fatigue. Grade 2 adverse events experienced by at least two patients that were assessed as being least remotely related to study drug were diarrhea, cervical lymphadenopathy, paresthesia and hypoesthesia (left facial numbness). One serious adverse event (SAE) occurred that was unrelated to vaccine. This patient developed a new sphenoid metastasis, attributed to progressive disease rather than vaccine administration.

### Biochemical/clinical Effect

The patients were followed only for a period of 8 weeks. PSA was measured at end of 4 weeks and 8 weeks (Table 2). Four patients had stable PSA through the 8 week study period (two patients had minimally decreased PSA levels at week 4, with one decrease sustained through week 8 (patients 002/009 and 003/011, respectively)). Patient 002–005 developed neurologic symptoms and MRI revealed a new sphenoid metastasis.

**Table 2:** Effect on PSA

**Patient ID**	**Baseline**	**Day 29 (± 2)**	**Day 57 (± 2)**
002–005	1353.10	1826.57	3150.50
002–007	89.00	91.22	108.53
002–008	108.50	190.23	265.74
002–009	1181.00	1141.30	1266.00
003-001	26.90	32.00	36.30
003-002	89.50	138.60	175.50
003-003	27.70	32.30	32.80
003–004	31.80	54.90	63.70
003–010	9.00	9.40	13.30
003–011	1424.00	1359.00	1268.00

### Immune response

**Figure 1:**
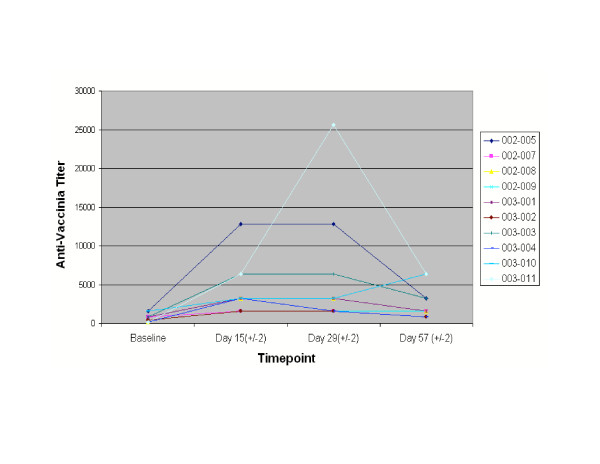
Effect of vaccination on anti-vaccinia antibody response: Antibody titers of patients are shown for baseline and days 15, 29, and 57.

All the patients were followed for immune response. None of the patients mounted any anti-PSA antibody response when evaluated by ELISA (data not shown). In contrast, anti-vaccinia titers increased in patients as measured by seroconversion at Day 15 (14 days post-vaccination; Table 3 and Figure 1). The mean anti-vaccinia titer at baseline was 700 (range 200–1600) likely due to prior smallpox vaccination. At the end of eight weeks the mean anti-vaccinia titer increased to 2720 (range 800–6400). One of the 10 patients developed an antibody response to fowlpox, as measured by seroconversion at Day 57 (28 days post-vaccination), (data not shown).

**Table 3:** Anti-Vaccinia Titers

**Patient ID**	**Baseline**	**Day 15 (± 2)**	**Day 29 (± 2)**	**Day 57 (± 2)**
002–005	1600	12800	12800	3200
002–007	800	1600	1600	1600
002–008	200	3200	3200	1600
002–009	400	1600	1600	1600
003-001	800	3200	3200	1600
003-002	400	1600	1600	800
003-003	800	6400	6400	3200
003–004	200	3200	1600	800
003–010	1600	3200	3200	6400
003–011	200	6400	25600	6400

## Discussion

The current study demonstrated that sequential vaccinations with PROSTVAC-V followed by PROSTVAC-F, including gene sequences for PSA and the three co-stimulatory molecules termed TRICOM, could be administered safely to men with androgen-independent prostate cancer. All patients demonstrated a rise in anti-vaccinia virus antibody titers without a detectable increase in anti-PSA titers. Four patients had stable post-vaccination PSA values at 8 week follow-up, with minimal decrease in two patients.

As a means to induce an immune response, the poxviruses are among the most commonly studied vectors for gene delivery [10]. Advantages of the poxviral vectors include the large size of the genomes, which have allowed as many as seven transgenes to be expressed in a single vaccinia virus vector, and greater immunogenicity of expressed proteins compared to native protein, attributed to the inflammatory response triggered against highly immunogenic viral proteins. PSA has also been commonly studied as an immunological target in prostate cancer [11,12]. Phase I clinical trials have evaluated the safety and biological effects of a vaccinia virus expressing human PSA in patients with prostate cancer without TRICOM [2-4]. Our prior study using fowlpox and vaccinia PSA-based vaccines without TRICOM in sequence demonstrated forty-one of sixty-four (64.1%) evaluable patients were free of PSA or clinical progression at 6 months with a trend favoring the arm with vaccinia followed by fowlpox vaccines. Although PSA declines of >50% were not noted, in one treatment arm, PSA doubling time changed from 4.5 months to 30.9 months supporting the hypothesis that the vaccine was slowing disease progression [6]. In the current study, patients were not assessed prior to therapy to determine changes in PSA velocity, since the main endpoint was to demonstrate safety of the PSA vaccine prime and boost approach with TRICOM for future studies.

A number of additional co-stimulatory molecules on antigen presenting cells have been identified including ICAM-1, B7.1, and LFA-3. Constructs using poxviral vectors (fowlpox and vaccinia) have been generated that contain this triad of co-stimulatory molecule transgenes, and have been given the designation TRICOM. Preclinical studies using TRICOM constructs have suggested that they are superior to those constructs that contain only one or two of the co-stimulatory molecules [7,8]. Studies have been completed combining CEA pox vaccines with TRICOM demonstrating safety of this approach. A Phase I clinical study has recently been completed employing vaccinia-CEA/TRICOM and fowlpox-CEA/TRICOM in a diversified prime and boost regimen, with most patients enrolled with GI malignancies [9]. The current study demonstrates that the PSA-based vaccine TRICOM has minimal toxicity. In contrast to previous trials using vaccinia virus or heterologous prime-boost vaccination with vaccinia followed by fowlpox viruses expressing PSA without TRICOM, which demonstrated effects on PSA velocity, this trial did not utilize repeated booster immunizations [4,6]. The current trial was designed to test the safety and feasibility of co-expressing PSA and TRICOM in patients with androgen-independent prostate cancer. A larger trial would be needed to determine clinical endpoints and should include on-going booster vaccinations in responding patients to determine long-term toxicity, biochemical response, and disease-free survival.

The current study also demonstrated increased anti-vaccinia antibody without any increase in anti-PSA antibody. In prior trials, an interferon-γ ELISPOT assay was used to monitor T cell responses against PSA in patients who were HLA-*A0201 positive, since there is a well defined HLA-A2-restricted peptide for *in vitro *targeting. Patients enrolled in the current trial were not HLA restricted and a validated T cell assay was not available. Therefore, we chose to measure the anti-PSA antibody response. Similar to previous trials, we failed to demonstrate an increase in anti-PSA antibody. In the randomized Eastern Cooperative Oncology Group Phase II study, there were no significant increases in anti-PSA antibody titers detected despite 46% of patients demonstrating an increase in PSA-reactive T-cells, and 45% biochemical progression-free survival at 19 months [6]. The reason for the absence of anti-PSA antibody response may relate to the sensitivity of the assay or to the presence of antigen-antibody complexes in the serum of men with elevated levels of PSA. Future studies with these vaccines may need to focus on the assessment of PSA-reactive T-cells and the determination of circulating immune complexes.

In summary, these data demonstrated the feasibility and safety of the PSA vaccine using a prime with vaccinia (PROSTVAC-V) followed by a boost with fowlpox (PROSTVAC-F) in which both vectors contain the gene sequences for the three co-stimulatory molecules, B7-1, ICAM-1, and LFA-3 (TRICOM) in men with androgen-independent prostate cancer. These data support further assessment of this vaccine approach in phase II and phase III clinical studies.
